# Maternal and infant death after probable vertical transmission of chikungunya virus in Brazil – case report

**DOI:** 10.1186/s12879-018-3243-1

**Published:** 2018-07-16

**Authors:** Rhaquel de Morais Alves Barbosa Oliveira, Francisca Kalline de Almeida Barreto, Ana Maria Peixoto Cabral Maia, Ileana Pitombeira Gomes, Adriana Rocha Simião, Rebeca Bandeira Barbosa, Adilina Soares Romeiro Rodrigues, Kilma Wanderley Lopes, Fernanda Montenegro de Carvalho Araújo, Regina Lúcia Sousa do Vale, John Washington Cavalcante, Luciano Pamplona de Góes Cavalcanti

**Affiliations:** 1 Collective Health, Federal University of Ceara (UFC), Fortaleza, CE Brazil; 2 Member of the Investigation Committee for arbovirus deaths, Fortaleza, CE Brazil; 3 Central Laboratory of Public Health of Ceará (LACEN), Fortaleza, CE Brazil; 4 Pathology, Federal University of Ceara (UFC), Fortaleza, CE Brazil; 5 Collective Health Departamento de Saude Comunitaria, Faculdade de Medicina, Federal University of Ceara (UFC), Fortaleza, CE Brazil

**Keywords:** Chikungunya virus, Arbovirus infection, Deaths, Vertical transmission

## Abstract

**Background:**

Chikungunya virus infection in neonates is relatively rare and can lead to death.

**Case presentation:**

We report the occurrence of the first death of a mother and child after probable vertical transmission of chikungunya virus in Brazil. A 28-year-old pregnant woman with hypertension presented with symptoms compatible with an arboviral disease at 34 weeks’ gestation. She developed preeclampsia with severe respiratory failure which resulted in the emergency cesarean section, and the patient died 12 days after the onset of symptoms. The pre-term newborn weighed 2535 g, with an Apgar score of 4/8. He was referred to the neonatal ICU with neutrophilia and thrombocytopenia, several seizure episodes, and hemorrhagic disorders, which resulted in death. Chikungunya IgM antibody was detected in the cerebrospinal fluid.

**Conclusions:**

We present the first documented maternal and neonatal death in Brazil after probable chikungunya infection during pregnancy.

## Background

The chikungunya virus is an arbovirus that has spread rapidly in recent years and has infected millions of people in more than 50 countries [1]. Main form of transmission is through the bite of infected mosquitoes of the genus *Aedes* [2]. However, there are reports of mother-to-child transmission [3–7]. In Brazil, during 2016 and 2017 over 300,000 cases of chikungunya were reported. In the State of Ceará, in the Northeast great region of Brazil, first cases occurred in 2015 culminating with a large outbreak in 2017 where the incidence was 1,520.3 cases/100.000 inhabitants and 157 attributable deaths were confirmed. The vertical transmission occurs mainly during the perinatal period and increases in the intrapartum period, with a transmission rate of approximately 50% [7], and can be fatal [7]. The disease is relatively rare in neonates and presents a diagnostic challenge. The clinical manifestations are varied, difficult to manage clinically, and carry a poor prognosis, which may result in death [6]. We report the occurrence of the first death of a mother and child after probable vertical transmission of chikungunya virus during an epidemic in the city of Fortaleza in northeastern Brazil in 2017.

## Cases

### Pregnant woman

A 28-year-old pregnant woman housewife at 34 weeks of gestation developed pruritus and pain in the lower belly on 4/4/2017. She had previously been diagnosed with hypertension and was being treated with methyldopa. She presented to the basic health unit that day and was prescribed an antihistamine. On 4/9/2017, she presented with fever, intense polyarthralgia, headache, arthritis in knees and ankles, cough, nausea, moderate abdominal pain and diarrhea (10 times). Although there was clinical suspicion of arbovirus infection, due to the occurrence of an epidemic of chikungunya, no laboratory tests were performed. On April 14, she presented to a health unit with pre-eclampsia, with a blood pressure (BP) measurement of 88/68 mmHg. She declined clinically, with persistent cough and dyspnea, and was transferred to a referral maternity hospital with severe dyspnea. Her oxygen saturation was measured at 70%, with a respiratory rate of 42 rpm and BP of 140/70 mmHg, and oxygen was supplemented with a Venturi mask. Obstetric evaluation revealed a fetal heart rate of 140 bpm, with cephalic presentation and an intact amniotic membrane. Venereal disease research laboratory (VDRL) and human immunodeficiency virus (HIV) tests were non-reactive. Thoracic radiographs revealed bilateral reticulonodular infiltrates. Medication for acute respiratory distress syndrome and mechanical ventilation were implemented. C-section with indication of intubation was recommended due to severe respiratory failure and peripartum cardiomyopathy. During hospitalization, furosemide, propofol, vasoactive drugs, Tamiflu, hydrocortisone, sedoanalgesia, insulin, meropenem, teicoplanin, calcium gluconate and sodium bicarbonate were administered. After the C-section (4/15/2017), she was transferred to the intensive care unit (ICU), and hemodialysis was performed. On the 2nd day of hospitalization (4/16/2017), the patient developed worsening of symptoms, with tachycardia, hyposaturation and fever, and hemodialysis was repeated. On the 3rd day (4/17/2017), the fever of 39.5 °C persisted, and she developed bradycardia after accidental extubation, which resolved after reintubation; a cardiopulmonary resuscitation cycle was performed. She also developed hypoglycemia and hypotension after use of vasoactive drugs at high doses. On the 4th day (4/18/2017), her condition worsened, with hemodynamic instability and cardiorespiratory arrest resulting in death.

### Newborn

The male infant was born of C-section to be consistent with general anesthesia on 4/15/2017. The results of the neonatal assessment included birth weight of 2535 g, length of 45 cm, head circumference of 33 cm, chest circumference of 30.5 cm, and an Apgar score of 4/8. At birth, the upper airways were aspirated with an orogastric catheter, which produced 4 ml of clear fluid, and the infant required external stimulation and positive pressure ventilation in the delivery room. The newborn was admitted to the neonatal ICU with Respiratory Distress Syndrome and cyanosis of the extremities; he was placed in a heated incubator, and nasal Continuous Positive Airway Pressure was instituted. On day 2, laboratory testing revealed hemoconcentration, thrombocytopenia and neutrophilia (Table 1). On the 4th day of life (4/19/2017), phototherapy for jaundice was implemented, and repeat laboratory testing revealed neutropenia and thrombocytopenia with elevated C-reactive protein (CRP). He returned to the neonatal ICU on 04/21/17 due to worsening thrombocytopenia, a significant increase in CRP, tachycardia, hyperthermia and mild respiratory distress. He was treated with an oxygen tent, antibiotic therapy and a platelet transfusion. On the 7th day (22/4/20/2017), the patient presented signs and symptoms suggestive of sepsis, such as bradycardia and apnea, requiring positive pressure ventilation until his condition stabilized. He presented lower gastrointestinal bleeding (30 ml of free blood). A transfusion of platelets and red blood cells was also administered. He had a seizure that responded well to anticonvulsant therapy. The next day (4/23/2017), the newborn developed severe pulmonary hemorrhage, anasarca, jaundice, seizures, hyposaturation and bradycardia. A nasopharyngeal swab sample was collected and was negative for influenza A and B, adenovirus, respiratory syncytial virus and parainfluenza viruses 1, 2 and 3. He was intubated, and mechanical ventilation was instituted, but resuscitation was unsuccessful. After his death, autopsy was performed due to the suspicion of arbovirus infection in the mother and the occurrence of a chikungunya epidemic in the city of Fortaleza. Biological samples were collected and sent to the reference laboratory. Serological tests and RT-PCR for dengue were negative in all the samples. Lung fragments were negative for influenza A and B. IgM for chikungunya virus was detected in the cerebrospinal fluid, and as he remained in the hospital all the time, he probably did not come in contact with mosquitoes. The cause of death was reported as digestive and pulmonary hemorrhage originating in the perinatal period. An autopsy revealed edema and congestion of the brain, with areas of ischemic necrosis of white and gray matter; marked alveolar hemorrhage; acute renal tubular necrosis; and splenic congestion. After an investigation by the Arbovirus Death Research Commission of the Department of Health of Ceará, the cases were confirmed as probable of the chikungunya by laboratory criteria in the neonate and by clinical epidemiological criteria in the mother. The baby’s father/husband had symptoms compatible with an arboviral diseases and IgM for chikungunya virus was detected in his blood sample, collected after the death of his wife.

**Table 1:** Laboratory results for the newborn that died of chikungunya virus infection in the 2017 epidemic in the city of Fortaleza, Brazil

Exams	4/17	4/19	4/21	4/22	Reference values
CRP	1,.4	39,.3	170,.4	132,.5	0,.25 – 0,.50
Hematocrit	56,.4	50,.3	45,.2	32,.4	34–40
Hemoglobin	20,.2	18,.1	15,.3	11,.1	11,.5–13,.5 g/dL
Leukocytes	6,230	5,140	13,950	22,740	5.,000–15.,000
Neutrophils	22,616	2,364	9,064	11,188	1.,500–8.,500
Lymphocytes	2,867	2,622	4,327	8,643	1.,500–7.,000
Platelets	169.,000	39.,000	7.,000	36.,000	140.,000–500.,000
Total Bilirubin	9,.54	6,.96	–	10,.25	0,.3–1,.2 mg/dl NB: < 24 h of life: 1,.4–8,.7 mg/dl < 48 h of life: 3,.4–11,.5 mg/dl 3 to 5 days of life: 1,.5–12,.0 mg/dl
Direct Bilirubin	0,.51	0,.84	–	8,.22	Up to 0,.2 mg/dl
Indirect Bilirubin	9,.03	6,.12	–	4,.03	Up to 1,.0 mg/dl
Sodium	141	–	–	130	135–145 mmol/L
Potassium	4	–	–	46	3,.5–5,.5 mmol/L
Calcium	9,.7	–	–	8,.4	8,.5–10,.2 mg/dL (2,.1–2,.5 mmol/L)
Magnesium	2,.1	–	–	2,.6	1,.7–2,.6 mg/dL (0,.7–1,.1 mmol/L)
Urea	26	–	–	8,.5	16–40 mg/dL
Creatinine	1,.2	–	–	2,.4	0,.6–1,.2 mg/dL

Legend: *CRP* C-reactive protein

Reference values [8]

## Discussion and conclusions

This is the first reported death of a mother and child after probable perinatal transmission of chikungunya virus in Brazil. In 2017, a chikungunya epidemic in the city of Fortaleza was recorded, with 71,478 cases and 141 deaths reported through the 48th epidemiological week; the peak of the epidemic coincided with the probable period of infection of the pregnant woman in this case report [9].

Chikungunya infection is currently a threat to maternal and child health [10]. However, the effects of maternal viremia in the prenatal period and the transmission mechanism for the newborn are not well-described [11].

Although confirmatory serological tests were not performed on the pregnant woman, she met the clinical and epidemiological criteria for chikungunya, and her case was confirmed after an investigation conducted by the experts of the arbovirus death investigation commission of the Health Secretariat [12]. Although for a confirmed case of chikungunya is necessary a PCR positive, a virus isolation positive or a serology test done on two samples showing a IgM/IgG conversion the clinical and laboratorial evidences, relatives with the same symptoms and the ongoing chikungunya epidemic support the confirmation of the case.

Chikungunya infection might possibly have been a contributing factor in her severe disease course, but could also have been coincident. During recent epidemics of chikungunya, the majority of patients hospitalized had preexisting diseases, and most of them experienced decompensation or exacerbation of these diseases, causing hospitalization [13]. Some studies have suggested that the presence of comorbidities, such as hypertension and diabetes, during chikungunya infection may worsen a patient’s clinical profile and increase the number of deaths [13–16]. While there is biological plausibility, the pathophysiology of this association remains elusive. Its consequences are still not fully clarified. Comprehensive investigations into the interactions between CHKV infection and hypertension are pivotal to elucidate the mechanistic basis of this association.

The neonate had a positive IgM to chikungunya result in cerebrospinal fluid, strongly suggesting of infection by vertical transmission. It should be noted that he remained hospitalized his entire life. Although there may have been mosquito exposure during the hospital stay, the chance of this occurring was very small. There was also the possibility of transmission during transfusions, but again, the chance of this occurring was very small [4, 5, 17].

In infected neonates, chikungunya symptoms usually develop between days 3 and 7 of life. A study of infected neonates showed that the most frequent laboratory abnormality was thrombocytopenia, which is associated with an elevated prothrombin time and disseminated intravascular coagulation [5]. Other studies have reported common symptoms such as high fever, irritability, erythematous maculopapular rashes, generalized hyperpigmentation, vesiculobullous lesions, swelling, neurological impairment (such as seizures), cardiac abnormalities, renal, respiratory or hepatic impairment and shock. The most common symptom of case reports of newborn infants with CHIKV by vertical transmission was thrombocytopenia [4, 5, 7, 18, 19]. In a study of 38 newborn infants with CHIKV vertical transmission, all of them presented thrombocytopenia, with 76% presenting mild thrombocytopenia (< 150–10^9^/L) and 12%, severe thrombocytopenia (< 50–10^9^/L) [4]. In another study conducted with 19 children diagnosed with neonatal CHIKV, 89.4% presented thrombocytopenia, and this result was associated with severe neonatal disease and led to the administration of multiple supportive interventions [5]. In addition to the afore mentioned symptoms, a study of eight infants showed that half of them exhibited hyperalgesia and respiratory distress, and two cases resulted in death [9]. Another study showed that early transmission of CHIKV (before 16 weeks of gestation) resulted in fetal death without malformations, with viral genome detected in the amniotic fluid, placenta and fetal brain [19]. When maternal infection occurs at the end of gestation, it is estimated that 12% of newborns are symptomatic, and most develop severe manifestations, such as meningoencephalitis [19–21]. A study during the intrapartum period indicated a vertical transmission rate of approximately 50% for viremic women, suggesting that this period is critical for transmission to the newborn [5].

In vertical transmission, the virus is inoculated directly into the fetal bloodstream, bypassing the usual dermal pathway to the lymphatic system and spreading directly into the circulatory system to infect organs, where viral replication continues [5, 7]. In addition, high maternal viral load, virus tropism to specific target organs, and neonatal host factors may contribute to disease severity [20].

An important point is that the delivery route does not contribute to infection, and infection is not avoided when delivery is by cesarean section, as evidenced in another study [18]. Although chikungunya virus is a significant maternal and child threat [9, 20], adequate research is lacking in South America [22–25].

For pregnant women in endemic areas, health professionals should maintain active surveillance for febrile conditions accompanied by arthralgia. There is also a risk of asymptomatic infection during pregnancy, which demands even greater attention at the time of evaluation [10]. Investigating suspected arbovirus deaths and understanding the role of infection in unfavorable outcomes is a challenge, especially in a location with more than 30 years of dengue virus, Zika virus and chikungunya virus transmission [26, 27].

While the infection of pregnant women with Zika virus appears to have a more severe effect on neonates during the first months of gestation, with chikungunya virus, infection is more severe when it occurs at the end of gestation. After these two deaths and the recent introduction of CHIKV in Brazil, along with the presence of several other arboviruses, further research is needed to understand chikungunya deaths and the factors associated with severe clinical cases and atypical manifestations.

## Abbreviations

BP: Blood Pressure
CHIKV: Chikungunya Virus
CRP: C-Reactive Protein
HC: head circumference
HIV: Human Immunodeficiency Virus
ICU: Intensive Care Unit
IgM: Imunoglobulina M
Nasal CPAP: Continuous Positive Airway Pressure
RT-PCR: Reverse Transcriptase Reaction
SDR: Respiratory Distress Syndrome
VDRL: Venereal Disease Research Laboratory
